# Progress in Applicability of Scoring Systems Based on Nutritional and Inflammatory Parameters for Ovarian Cancer

**DOI:** 10.3389/fnut.2022.809091

**Published:** 2022-04-08

**Authors:** Juan Mu, Yue Wu, Chen Jiang, Linjuan Cai, Dake Li, Jian Cao

**Affiliations:** ^1^Department of Nutrition, Nanjing Maternity and Child Health Care Hospital, Women’s Hospital of Nanjing Medical University, Nanjing, China; ^2^Department of Gynecology, Nanjing Maternity and Child Health Care Hospital, Women’s Hospital of Nanjing Medical University, Nanjing, China

**Keywords:** nutritional support, efficacy evaluation, nutritional screening, inflammatory parameters, ovarian cancer

## Abstract

Ovarian cancer is a malignancy that seriously endangers women’s health; its case fatality rate ranks first among the gynecological malignancies. The status of nutrition of ovarian cancer patients is related to their prognoses. Thus, it is important to evaluate, monitor, and improve the nutritional status of ovarian cancer patients during their treatment. Currently, there are several tools for examining malnutrition and nutritional assessment, including NRI (nutrition risk index), PG-SGA (patient-generated subjective global assessment), and NRS 2002 (nutritional risk screening 2002). In addition to malnutrition risk examination and related assessment tools, the evaluation of muscle mass, C-reactive protein, lymphocytes, and other inflammation status indicators, such as neutrophils to lymphocytes ratio, lymphocyte-to-monocyte ratio, and C-reactive protein-albumin ratio, is of great importance. The nutritional status of ovarian cancer patients undergoing surgery affects their postoperative complications and survival rates. Accurate evaluation of perioperative nutrition in ovarian cancer patients is crucial in clinical settings. An intelligent nutritional diagnosis can be developed based on the results of its systematic and comprehensive assessment, which would lay a foundation for the implementation of personalized and precise nutritional therapy.

## Introduction

Ovarian cancer is a gynecological malignancy associated with the highest fatality rate. Approximately 70% of ovarian cancer patients are diagnosed at the advanced clinical stages on their first visit to the doctor. Patients often report ventosity, abdominal pain, intestinal obstruction, decreased appetite, and nausea, which in turn affects their nutritional intake (1). Studies show that the malnutrition incidence among ovarian cancer patients is significantly higher than that in other gynecological diseases; the median survival time of malnourished ovarian cancer patients is also shorter than the of well-nourished patients (2). Multi-mechanism underlies the occurrence of malnutrition and cachexia in ovarian cancer patients. The tumor itself causes metabolic disorders in the body as catabolism is greater than anabolism. Patients with advanced ovarian cancer are prone to malignant intestinal obstruction and gastrointestinal metastasis; tumor enlargement can also lead to mechanical obstruction of the gastrointestinal tract (3). Some non-specific symptoms caused by huge solid tumors and ascites trigger the loss of appetite. In addition, the activation of inflammatory responses increases the synthesis and the entry of pro-inflammatory factors in the blood. Secretion of acute proteins [such as C-reactive protein (CRP)] can promote tumor cell proliferation and support the growth of primary tumors, leading to the formation of a microenvironment conducive for metastasis and further secondary metastasis (4). At the same time, due to the high immunosuppressive microenvironment, multiple types of cells interact with inflammatory factors to further promote the formation of tumors. When patients are undernourished, they have a low tolerance to surgery, show insensitivity toward radiotherapy and chemotherapy, and decreased immune function, which predisposes them to secondary infections. Therefore, clinical nutritional therapy is of great significance for cancer patients. In addition to traditional nutrition risk screening, clinical nutrition assessment should consider the assessment of muscle mass and function and evaluation of systemic inflammatory state (5). This review aimed to discuss the guiding significance of the scoring system based on nutritional and inflammatory parameters in the prognosis of ovarian cancer. It may provide a reference for further clinical evaluation and development of individualized treatment strategies.

## Nutritional Indicators

### Nutrition Risk Index

Nutrition risk index (NRI) proposed by the Parenteral Nutrition Research Collaborative Group of the American Veterans Association in 1991, is used to examine the effects of total parenteral nutritional support for patients before major clinical abdominal surgery and thoracic surgery. The primary reference indicators are the percentage of weight loss and serum albumin level (6). A study included 660 patients who had undergone radical gastrectomy showed that malnutrition was significantly associated with postoperative wound complications after gastrectomy. NRI on the fifth day post-surgery could predict postoperative wound complications after gastrectomy (7). Yim et al. (8) conducted an NRI-based study in 213 patients with ovarian cancer. Among them, 78% of the patients had low-to-mild nutritional risk, while the other 22% were in the moderate-to-severe nutritional risk group. The 5-year overall survival (OS) rate in ovarian cancer patients with moderate-to-severe nutritional risk (45.3%) was significantly lower as compared to those at low-to-mild nutritional risk (64.0%), respectively (*P* = 0.024); the progression-free survival (PFS) period was substantially shortened in the moderate-to-severe nutritional risk group (15 vs. 28 months, *P* = 0.011). Yoon et al. (9) studied the applicability of NRI to assess the relationship between survival rate and nutritional factors before and after chemotherapy. A total of 212 patients in stage III/IV of ovarian cancer who had undergone surgery along with six courses of chemotherapy with cisplatin and paclitaxel, were enrolled. The results showed that NRI was significantly related to survival time; the survival time of patients with moderate-to-severe malnutrition before chemotherapy (48 months) was significantly shorter as compared to those with mild-to-moderate malnutrition (80 months). The relationship between NRI and the OS rate after treatment was in line with the previous studies. The relative risk of death in patients with moderate-to-severe malnutrition was 3.6 times greater as compared to those with mild-to-moderate malnutrition. Compared with other composite indicators, NRI is simple, easy to use, and has better sensitivity and specificity. However, its main disadvantage is the pre-requisite data of the patient’s current and past weights. If the patient develops edema due to the disease, the NRI measurement is affected. In addition, owing to the effect of stress on serum albumin concentration, the use of NRI screening is limited in clinical settings.

### Nutritional Risk Screening 2002

The Danish Parenteral and Enteral Nutrition Association has developed NRS 2002 (nutritional risk screening 2002), the first nutritional risk screening tool that relies on evidence-based medicine. It is also recommended by the European Society for Clinical Nutrition and Metabolism (ESPEN).

The core indicators selected to reflect the nutritional risk were derived based on 128 randomized controlled trials (RCTs) (10). It is suitable for nutritional risk evaluation of inpatients and is not only simple but also easy to implement. The scoring method for patients is divided into two parts according to the nutritional status and disease severity; each part is further divided into four levels. When the total score ≥3, patients are “at nutritional risk.” NRS 2002 has good sensitivity and specificity. Bargetzi reports that NRS is closely related to the patient’s 180-day mortality rate. Each point increase in the patient’s NRS score is associated with a 37% increase in the risk of all-cause death in cancer patients within 180 days. In addition, NRS is associated with the composite endpoint for adverse outcomes, the average hospitalization time, impairment in quality of life, and functional decline (11). NRS 2002 is mainly used for the evaluation of patients with gastric cancer and esophageal cancer having a high incidence of malnutrition (12, 13). In addition, NRS 2002 nutritional risk screening tool can help in the identification of malnourished patients who need to be given different nutritional support. It provides theoretical support for the formulation of personalized treatment plans and has good guiding and predictive effects on the nutritional screening of patients with colorectal cancer (14). However, NRS is rarely used in patients with gynecological tumors. Hertlein et al. (15), using NRS 2002, performed nutritional risk screening for 47 patients with ovarian cancer and found that 70.2% (33 cases) of total patients were at nutritional risk, which is, NRS 2002 score was ≥3 points.

Nutritional risk is also related to the incidence of surgical complications and duration of hospital stay. Another study by Hertlein et al. (16) shows that perioperative immune nutrition supplementation in patients with malnourished ovarian cancer with an NRS 2002 score of ≥3 does not significantly improve the complication rate and hospital stay, but it can reduce complications due to infections. Deficiencies were also found during NRS 2002 application. During the screening process, for patients with deviations in their weight change estimates and dietary intake, or those who did not answer the questions, the results were not accurate. Kyle et al. analyzed the relationship between the NRS 2002 score and the prognoses of 995 inpatients and reported that NRS 2002 could not objectively and accurately reflect the nutritional status of some patients (17). The main reason is that NRS 2002 integrates the patient’s nutritional status, disease severity, age along with other factors. Thus, there are more subjective components and individual indicators have significant weights (18). NRS 2002, which gives greater consideration to the complications in nutrition-related diseases, still does not solve the problem of the lack of a unified standard for the evaluation of the nutritional status in patients. Further, its utility for ovarian cancer is less explored.

### Subjective Global Assessment and Patient-Generated Subjective Global Assessment

Subjective global assessment (SGA), recommended by the American Society for Parenteral and Enteral Nutrition (AND/ASPEN), is a screening tool that includes detailed medical history and physical evaluation parameters (19). Patient-generated subjective global assessment (PG-SGA) is based on SGA, which consists of two parts: patient self-assessment and medical staff assessment. The self-assessment part further includes four aspects, which include, food intake, weight, symptoms, activity, and physical function. If the PG-SGA score is greater than or equal to 9 points, a comprehensive assessment should be performed, followed by nutritional intervention; anti-tumor treatment should be suspended. Gupta et al. (20) performed an SGA-based evaluation of 98 ovarian cancer patients and found that 47% of patients were A-graded, which implied having good nutrition; 29% were B-graded, which implied mild-to-moderate malnutrition, and 24% were C-graded, indicative of severe malnutrition. At 3 months, the median survival time in the grade A group was significantly longer than that in the B and C groups (19.9 vs. 3.7 months, *P* < 0.001). The patient survival rate due to improved nutritional status after 3 months was significantly higher than that of patients with worsened nutritional status. These findings were independent of age, diagnosis time, treatment history, and CA125. Chantragawee (21) reports that as compared to endometrial cancer and cervical cancer, malnutrition is more common in patients with ovarian cancer based on PG-SGA. Phippen reports that (22) patients who experience febrile neutropenia (FN) have a higher PG-SGA score, and it may be a reasonable predictor of FN in patients with gynecological malignancies receiving multi-drug primary chemotherapy. It may also be beneficial for preventive GCSF. Das et al. (23) used PG-SGA to assess the status of nutrition of 60 patients with gynecological malignancies. A total of 88.33% of patients with gynecological tumors had a certain degree of malnutrition or were at risk of malnutrition. Approximately 5% weight loss in the preceding month could replace the comprehensive score PG-SGA in triage patients. Laky et al. (24) used the SGA and PG-SGA scales for the nutritional assessment of 194 patients with different gynecological tumors. The incidence of malnutrition in ovarian cancer patients was the highest, estimated to be 67%. They found that the evaluation results of SGA and PG-SGA were very similar. However, SGA could not accurately reflect the changes in acute nutritional status and lacked evidential support for screening and clinical outcomes. However, PG-SGA is significantly related to objective and subjective parameters and is widely considered as a relevant method for examining the nutritional status among patients in clinical settings (25).

### Prognostic Nutritional Index

Prognostic nutritional index (PNI) is used for the assessment of the nutritional status among patients who have undergone surgery, predicting surgical risks, and for prognostic judgments. It was first established by Onodera et al. (26), a Japanese scholar. Originally, PNI was used for the evaluation of the nutrition and immune status of patients undergoing gastrointestinal surgery. It is determined according to the lymphocyte count and level of serum albumin in the peripheral blood. In recent years, it has been used as a new indicator for prognostic judgment of patients with gastrointestinal malignant tumors, gynecological tumors, and lung cancer (27). PNI reflects preoperative malnutrition and is used to predict the incidence of postoperative complications. It is also a prognostic predictor for the long-term progression of various malignant tumors. Yoshikawa et al. (28) used PNI 46.5 as the critical value for ovarian clear cell carcinoma patients. The OS of the patients in the PNI high group was significantly longer than those in the PNI low group. Multivariate analysis indicated that high PNI could be an important independent potential predictive prognostic factor for a good prognosis. The disease-free survival rate of the two groups was not abnormal, but the postrecurrence survival was significantly higher in the high-PNI group than in the low-PNI group [hazard ratio (HR) = 6.43; 95% CI, 1.09–121.64 months, *P* = 0.0383]. Komura et al. (29) retrospectively analyzed data of 308 patients in stages I–IV of epithelial ovarian cancer. In early ovarian cancer, PNI = 44.7 was used as the cut-off value, and in advanced ovarian cancer, PNI = 42.9 was the threshold. In early ovarian cancer patients, reduced PNI was not significantly correlated with PFS and disease-related survival. However, multivariate analysis for advanced ovarian cancer showed that low PNI could be an independent predictive risk factor for PFS and disease-related survival. In addition, they found that for the prediction of disease-specific survival in patients with epithelial ovarian cancer, the PNI before treatment was a better indicator than the platelet count. Although thrombocytosis before treatment is used as an independent factor for poor prognoses in patients with epithelial ovarian cancer, it usually reflects lower PNI, and no prognostic information is available when adjusting for the PNI values (30). Feng et al. (31) used PNI = 46.2 as the critical value and showed that low preoperative PNI was correlated with the FIGO stage progression, elevated CA125 level, extensive presence of ascites, residual tumors, and platinum resistance. In multivariate analysis, PNI as a continuous variable was an independent predictor of OS. PNI is a validated prognostic predictive parameter for high-grade serous ovarian cancer (HGSC). Miao et al. (32) used PNI = 45 as the cut-off value and found that the AUC of PNI-predicted platinum resistance was 0.688; the sensitivity was 62.50%, and the specificity was 83.47%. The median PFS of patients with a lower PNI (<45) was 12 months (95% CI, 10.62–13.38 months), whereas the median PFS of patients with a higher PNI (≥45) was 23 months (95% CI, 18.03–27.97 months). PFS and OS in the low-PNI group were significantly lower than those in the high-PNI group (both *P* < 0.001). Multivariate analysis showed that PNI < 45 was an independent risk factor for PFS and OS outcomes. Zhang et al. (33) retrospectively analyzed the data of 237 patients with epithelial ovarian cancer using PNI = 47.2 as the cut-off value. They found that the PFS in the low PNI group was significantly lower than in the high PNI group. For low and high PNI groups of platinum-sensitive patients, PFS was 49.4 and 28.9 months (*P* < 0.001), respectively, and OS was 55.7 and 82.7 months (*P* < 0.001), respectively. However, there were no statistically significant differences in PFS and OS between the two groups of patients demonstrating platinum resistance. The efficacy of PNI in predicting OS and resistance to platinum was higher than CA125. Thus, PNI, owing to its high efficiency and simplicity, has been widely used, in evaluating the pre-treatment status of patients with various malignancies. Although some studies report that PNI is related to the prognosis of ovarian malignant tumors, these studies have some limitations that need to be addressed. The sample size in single-center retrospective studies is limited, and whether the same conclusion applies to different populations and different cancer types needs to be investigated in the future. Currently, there is no uniform standard for the best cut-off value of PNI. Differences in the selection criteria and method selection also need to be addressed (34).

### Skeletal Muscle Index

Skeletal muscle index (SMI) is widely used to evaluate sarcopenia. It is measured as the total area of all skeletal muscles (psoas major, erector spinae, quadratus lumborum, transverse abdominis, extra-abdominal; the total area of the oblique muscles and internal oblique muscles) divided by the height squared (35). The area of skeletal muscle is evaluated using several methods, such as bioelectrical impedance analysis (BIA), dual-energy X-ray absorptiometry (DXA), and CT scan imaging. Considering the clinical practicality and economic factors, currently, the CT imaging method is being widely used (36). The third lumbar spine SMI is widely used for nutritional assessment and in the assessment of tumor prognosis. However, there is no uniform standard cut-off value (37). Staley, using SMI 41 as the cut-off value, evaluated 201 patients with epithelial ovarian cancer and found that sarcopenia was not associated with poor survival outcomes or chemotherapy toxicity. Prospective studies in the future should focus on interventions to prevent or reverse sarcopenia, improve the survival, performance status, and quality of life of patients with ovarian cancer (38). Kim et al. analyzed the data of 179 patients in stages III–IV of HGSC using SMI 39 as the cut-off value. They found that the PFS and OS of patients in the sarcopenia and control groups were similar. In the subgroup analysis of the sarcopenia group, the OS for high fat-to-muscle ratio (FMR) group patients was significantly lower than that in the low FMR group. High FMR was an independent prognostic factor for poor OS in the sarcopenia group (5-year survival rate, 44.7 vs. 80.0%; *P* = 0.046) (39). Ataseven used SMI along with muscle attenuation [MA; Hounsfield units (HU)] and analyzed 323 cases of advanced epithelial ovarian cancer. They found no statistically significant differences in PFS and OS between the patients in the sarcopenia and control groups. However, low MA was correlated significantly with OS, particularly in patients exhibiting residual tumors. MA assessment can be used for risk stratification after tumor reduction (40). Rutten retrospectively analyzed 216 patients with ovarian cancer who underwent primary debulking surgery (PDS) treatment using SMI 38.73 as the threshold. Patients with sarcopenia had a significant survival disadvantage. However, the skeletal muscle reduction could not predict OS or other grave complications in ovarian cancer patients (41). Yoshino assessed the skeletal muscle area (SMA) at the third lumbar vertebrae in 60 patients at stage III/IV EOC who underwent induction chemotherapy (IC). The cut-off value of SMA-to-SMA ratio (SMAR) before and after IC was determined; SMAR critical value was 0.96 and low SMAR could predict poor prognosis of IC in patients with advanced EOC (42). Ubachs found that SMI reduction in ovarian cancer patients in stage III undergoing neoadjuvant chemotherapy (NACT) was not associated with a worsening prognosis. However, there was a positive correlation between SMI and adverse events (43). Skeletal muscle depletion, which affects the patients’ ability to receive treatment. A total of 893 adverse events (70.6%) were reported preoperatively in the decreased SMI group, compared with 372 events (29.4%) in the stable/increased SMI group (*P* = 0.008). The percentage of grade 3–4 events (such as pulmonary embolisms, coagulation disorders with clinical symptoms, gastrointestinal function significantly changed) in the reduced SMI group (5.3%) was higher than that in the stable SMI group or the elevated SMI group (2.6%). Huang conducted a retrospective analysis of 139 ovarian cancer patients in stage III and found that, during treatment, SMI significantly reduced and was independently correlated with poor OS in stage III EOC patients who received adjuvant platinum-based chemotherapy and PDS. The modified Glasgow prognostic score (mGPS) could be a potential predictor of SMI decline during treatment (44). SMI includes skeletal muscles at the caudal level of the third lumbar vertebra from the CT images. This plane has several muscles and is a complex region to perform measurements. Large area inclusion may increase measurement errors. The current knowledge on SMI and ovarian cancer is based on retrospective studies. Thus, future prospective studies will be of great significance for prognostic prediction of skeletal muscle state (45).

### Psoas Muscle Index and Psoas Muscle Volume

Psoas muscle index (PMI) is the value obtained from the measurement of the cross-sectional area of the psoas major muscle on either side. It is calculated as the sum of the area divided by the square of the height. Some studies have pointed out that PMI and SMI are not well comparable, and the SMI measured by CT cannot be used interchangeably (46). We speculate that PMI is easier to measure and calculate than SMI. The quality of psoas major muscle, which maintains the stability of body posture and conducts the strength of upper and lower limbs, is easily affected by the patient’s own nutritional status and daily activities. The psoas major muscle is located in the abdominal cavity and has a fixed position. In recent years, studies from abroad show that indicators based on the area of the psoas muscle are significantly related to the prognosis of abdominal surgery, and also represent to a certain extent, the skeletal muscle content of the whole body (47). Yoshikawa evaluated the data of 72 patients suffering from epithelial ovarian cancer and undergoing combination therapy with paclitaxel and carboplatin; PMI of 5.4 was the critical value. Compared to the patients with lower PMI, the OS of patients with higher PMI significantly improved. Multivariate analysis of OS showed that low PMI was an independent unfavorable prognostic factor and that PMI may provide a potential prognostic biomarker for epithelial ovarian cancer patients (48). Matsubara et al. enrolled 92 epithelial ovarian cancer patients and calculated the psoas muscle volume (PV) based on their three-dimensional CT (3D-CT) scans. Patients with low PV had significantly worse PFS and OS; PV was found to be better than SMA and psoas area (PA) in predicting prognosis (49). Psoas index (PI) is the main cross-sectional area of the psoas muscle divided by the height squared. Yoshikawa evaluated the median PI of 76 patients with ovarian cancer undergoing first-line chemotherapy. Compared with patients having high PI, those with low PI were more likely to develop peripheral neuropathy (32 vs. 11%; *P* = 0.047). The PI value was independent of other toxicities such as neutropenia and thrombocytopenia. Thus, the median PMI can serve as a potential predictive biomarker for toxicity in ovarian cancer patients (50). Rutten speculates that changes in the psoas muscle area cannot represent alterations in the total muscle area, and that total skeletal muscle cannot be used as a substitute for predicting the survival of patients with ovarian cancer (51). Taken together, studies based on psoas major muscle and ovarian cancer need further prospective validation. The development of a unified evaluation system would be more valuable for studying the prognosis of ovarian cancer. We presented a comparison of commonly used nutritional indicators in cancer patients (Table 1).

**TABLE 1:** Comparison of commonly used nutritional indicators for cancer patients.

	NRI	NRS 2002	SGA	PG-SGA	PNI	SMI	PMI	PV
Age	×	✓	×	×	×	×	×	×
BMI	✓	✓	✓	✓	×	×	×	×
Involuntary weight loss	✓	✓	✓	✓	×	×	×	×
Diet-related symptoms	×	×	✓	✓	×	×	×	×
Dietary changes	×	✓	✓	✓	×	×	×	×
Physical activity	×	×	✓	✓	×	×	×	×
Disease severity	×	✓		✓	×	×	×	×
Physical examination	×	×	✓	✓	✓	✓	✓	✓
The laboratory indicators	✓	×	×	×	✓	×	×	×

## Inflammatory Indicators

### Neutrophil-to-Lymphocyte Ratio

Neutrophil-to-lymphocyte ratio (NLR), an inflammation index, reflects the dynamic balance between neutrophils and lymphocytes, and comprehensively represents the patient’s immune status. Recent studies report that the prognosis of malignant tumors is closely related to clinicopathological signs, and that chronic inflammation plays a crucial role in tumor invasion and metastasis (52). NLR can predict the prognosis of several solid tumors, including lung cancer, breast cancer, and ovarian cancer (53, 54). Medina Fernández et al. (55) included 122 advanced ovarian cancer patients and found that during a concurrent infection, CRP peaked at 48 h, while NLR peaked at 24 h; NLR was more effective for predicting infection-related complications. Zhou et al. (56) retrospectively analyzed 370 epithelial ovarian cancer cases in FIGO III using NLR = 3.08 as the cut-off value and found that PFS and OS of patients in the NLR high group were substantially lower than those in the NLR low group (*P* < 0.05); NLR and PLT could jointly predict the OS. Feng et al. (57) through factor analysis, reported that high NLR was only related to PFS. Salman et al. (58) found that between the NLR ≥ 6.0 and the NLR < 6.0 groups, there was no statistically significant difference in the rates of optimal debulking. However, there was a significant correlation between high NLR and OS. Williams et al. (59) reports that high NLR values are correlated with advanced tumor stage and higher grade, bilateral adnexal masses, presence of ascites, and related risk factors, including greater height, Jewish ethnicity, family history of cancer, more ovulation cycles, and use of talcum powder in premenopausal women. In patients at FIGO stages IIIC and IV, who underwent NACT, Sanna prospectively evaluated the dynamic changes in NLR for patients with HGS advanced epithelial ovarian cancer. The decrease in NLR after three cycles was significantly associated with a better response to NACT; the PFS was significantly higher as compared to patients whose NLR value increased after three cycles of NACT. Thus, the changes in NLR during treatment can be used as a response predictor for NACT in HGS advanced ovarian cancer patients, which means that NLR was elevated and chemotherapy was less effective (60). Marchetti performed retrospective analyses of the NLR and BRCA gene status of 39 epithelial ovarian cancer patients; regardless of BRCA mutant or wild-type, the median progression free survival in the low NLR group was longer than that in the NLR group. Thus, NLR is a validated prognostic marker for OC patients and is independent of the BRCA mutation status (61). Wu et al. collected data for 262 ovarian cancer patients; among them, 258 patients had benign ovarian cancer. A total of 232 healthy controls were also included. The derived neutrophil-to-lymphocyte ratio (dNLR) was evaluated based on parameters of whole blood cells. dNLR was substantially different between ovarian cancer, benign ovarian disease, and the healthy control groups. It was positively correlated with ovarian cancer staging and CA125 (all *P* < 0.001). Thus, dNLR can be used as an effective indicator to differentiate ovarian cancer from benign disease (62). Taken together, NLR is closely associated with the clinical characteristics of ovarian serous epithelial cancer, including FIGO staging, degree of differentiation, and tumor markers. Thus, NLR has a high value for evaluating the prognosis of patients.

### Platelet-to-Lymphocyte Ratio

Platelet-to-lymphocyte ratio (PLR) is the ratio of platelets to lymphocytes. Studies show that platelets perform the function of sensing, monitoring, and transmitting information. Tumor cells cause loss of vascular endothelium, activation of platelets, and formation of platelet-vascular wall-tumor cell interactions. It may be related to the balance between platelet-dependent pro-tumor inflammatory response and lymphocyte-mediated anti-tumor immune response in the tumor microenvironment (63). The release of various inflammatory mediators can induce an increase in the platelet number. Activated platelets secrete platelet-derived growth factor (PDGF), platelet-activating factor (PAF), and vascular endothelial growth factor (VEGF) along with several other cytokines to promote the formation of tumor-related blood vessels and the degradation of extracellular matrix, which in turn enhance tumor growth and distant metastasis (64). PLR is closely related to the recurrence and survival cycle of malignant tumors (65). Asher et al. (66) retrospectively analyzed data from 235 patients with ovarian cancer and found that the OS of patients with PLR <300 and PLR ≥300 were 14.5 and 37.4 months, respectively. Multivariate analysis suggested that high PLR was an independent prognostic factor for ovarian cancer. Badora-Rybicka (67) retrospectively analyzed 315 cases of ovarian cancer. Similarly, high PLR was an independent predictive risk factor for PFS, however, it did not affect OS. Raungkaewmanee et al. (68) found that for PLR ≥200 the AUC of FIGO staging was 0.66, sensitivity was 72.7%, and specificity was 65.7%. The patients whose PLR >200 showed shorter PFS and OS. Single-factor analysis indicated that high PLR was a potential risk factor for OS. Taken together, PLR has potential predictive clinical value in advanced diseases. Compared with thrombocytosis or NLR, PLR is a better prognostic indicator for EOC patients. Zhang et al. (69) performed a multivariate analysis with PLR = 203 as the cut-off value. Unlike CA125, NLR, fibrinogen, CRP, and albumin levels, PLR was an independent risk factor for PFS. Thus, for prognostic prediction of ovarian cancer, preoperative PLR is better than CA125, NLR, fibrinogen, CRP, and albumin levels. Zhao et al. performed a meta-analysis of 13 studies consisting of 3,467 patients with ovarian cancer and found that those with PLR ≥200 had shorter OS and PFS. Therefore, high PLR is correlated to poor prognosis (70).

### Lymphocyte-to-Monocyte Ratio

Lymphocytes and monocytes are the key immune cell types mediating the inflammatory response. Lymphocyte-to-monocyte ratio (LMR), a combination of tumor-related inflammatory cells, is related to the prognoses of several tumors (71). Existing immunological studies show that lymphocytes, forming the core of the body’s immune response, participate in cellular immunity and humoral immunity. Among them, the T lymphocytes perform the functions of anti-tumor cells and exhibit anti-infection and anti-allogeneic effects. Several studies report an increase in T-lymphocytes in the peripheral blood of ovarian cancer patients (72, 73). Monocytes can produce a variety of cytokines and chemokines, which in turn, promote the occurrence and progression of tumors by immunosuppressive effects and stimulation of tumor angiogenesis. Monocytes can also produce tumor-associated macrophages (74). TAMs can promote the efficacy of tumor angiogenesis by secreting angiogenic factors and regulate the degradation of the extracellular matrix through enzymes and inhibitors, beneficial for tumor migration and progression. However, TAMs also simultaneously exert anti-tumor effects. Their prognostic influence is the result of the interaction between the tumor-promoting and anti-tumor effects. Therefore, the peripheral blood lymphocyte count can reflect a certain degree, the anti-tumor immune response to ovarian cancer. A decrease in the peripheral blood lymphocyte count may lead to a decline in the tumor immune response, thereby promoting tumor progression and metastasis. Monocytes derived from inflammatory chemokines and cytokines can promote tumor progression (75). Yang (76) evaluated the clinical data of a total of 364 newly diagnosed epithelial ovarian cancer patients. The best cut-off for LMR to predict the survival of patients with epithelial ovarian cancer was estimated at 3.84; the median follow-up time was 37 months. The results of multivariate analysis showed that postoperative FIGO stages III–IV, poorly differentiated tumor grade, presence of lymph node metastasis, absence of postoperative adjuvant treatment, and LMR ≤ 3.84, were independent risk factors affecting PFS and OS in epithelial ovarian cancer patients. Kwon et al. (77) included the clinical data of 109 ovarian clear cell carcinoma patients. Using an LMR cut-off of 4.2, high LMR was found to be significantly correlated to high 5-year PFS and OS. FIGO staging, residual disease, and platinum remission were independent prognostic factors for PFS, while FIGO staging, residual disease, platinum remission, and LMR were independent prognostic factors for OS according to the results of multivariate analysis. Thus, LMR is the most reliable independent factor for the OS prognosis in ovarian clear cell carcinoma patients. According to the data for the entire cohort, the optimal LMR threshold selected based on PFS and OS ROC curves was 2.07. Eo et al. (78) collected clinical data of 234 epithelial ovarian cancer patients. The 5-year OS rates in the LMR low and the LMR high groups were 42.2 and 67.2%, respectively; the 5-year PFS rates in the two groups were 40.0 and 62.5%, respectively. According to the multivariate analysis, the most important prognostic factors that influenced PFS were age, FIGO stage, and tumor antigen 125 level; LMR was the most valuable prognostic factor for OS prediction. A meta-analysis of LMR for ovarian cancer patients by Gong confirms that low LMR is associated with worse OS and PFS; it is also significantly related to G2/G3 classification, III–IV staging, CA125, and malignant ascites. The author also discussed elaborately the inconsistency in cut-off values of LMR in the included studies, and their retrospective designs, particularly in Asia. These may result in bias and need to be addressed in future investigations (79).

### C-Reactive Protein-Albumin Ratio and Glasgow Prognostic Score

C-reactive protein-albumin ratio (CAR) is a recently developed indicator that comprehensively evaluates the level of inflammation and nutritional status of the patient. It is related to the prognosis of several tumors. Tumor-related inflammation plays an important role in the infiltration, proliferation, tumor progression, and metastasis of tumor cells (80). CRP is synthesized by the liver in response to infection, inflammation, and tissue damage, and is regulated by several cytokines. CRP is a highly specific marker of systemic inflammation (81). Patients with ovarian cancer often show elevated serum CRP levels, which indicates that there is a chronic inflammatory response to the progression of ovarian cancer (82, 83). In clinical settings, serum albumin level is primarily used for the assessment of the nutritional status of patients. The malnutrition of patients caused by tumors and the host response to these tumors can alter albumin levels. Decreased albumin levels can lead to undernourishment in patients and affect their prognoses. Komura et al. (84) retrospectively analyzed the data from 308 epithelial ovarian cancer patients and found that regardless of the clinical-stage or the rate of reductive surgery, elevated CRP/Alb remained an independent predictor of short-term disease-specific survival. CRP/Alb was better than CRP for the prediction of disease-specific survival in EOC patients (HR = 1.96; 95% CI, 1.10–3.57; *P* = 0.0221). Liu et al. (85) using CRP/Alb = 0.68 as the critical value, found that elevated CRP/Alb was associated with advanced stage, residual tumor, ascites, elevated CA-125 levels, Glasgow prognostic score (GPS), and mGPS. CRP/Alb was an independent prognostic factor for OS. The AUC values for CRP/Alb at 1-, 3-, and 5-years were higher than those for GPS, mGPS, and PNI.

The GPS scoring system combines CRP and AIb levels. When CRP > 10 mg/L and AIb < 35 g/L, the GPS is considered as two points. When one indicator is abnormal, it is one point. Both indicators are normally scored as 0. Sharma et al. (86) retrospectively analyzed data of 154 ovarian cancer patients in advanced stage and found that GPS was an independent factor of the OS rate (*P* < 0.05). The higher the GPS score, the worse was the prognosis. Omichi et al. (87) analyzed the data of 216 patients with epithelial ovarian cancer and found that in all the stages of ovarian cancer, PFS and OS were shorter when the GPS score was 2 points as compared to 0 and 1 point. According to multivariate analysis, a high GPS score was determined as an independent risk factor for recurrence and OS in all stages of ovarian cancer, regardless of the histological grade. Zhu et al. (88) retrospectively analyzed 672 advanced ovarian cancer patient data and found that high GPS scores were associated significantly with postoperative residual tumor size (*P* = 0.007), histological grade (*P* = 0.001), and histological type of the tumor (*P* = 0.013). High GPS scores reflected a low rate of complete remission post NACT, and the OS rate and disease-free survival time were substantially shortened (all *P* < 0.001). We presented a comparison of commonly used inflammation indicators in cancer patients (Table 2).

**TABLE 2:** Comparison of commonly used inflammation indicators for cancer patients.

	NLR	PLR	LMR	CAR	GPS
Neutrophils	✓	×	×	×	×
Lymphocytes	✓	✓	✓	×	×
Platelets	×	✓		×	×
Monocyte	×	×	✓	×	×
C-reactive protein				✓	✓
Albumin	×	×	×	✓	✓

## Conclusion

In summary, the clinical significance of nutritional screening tools is not only for the evaluation of the preoperative nutritional status, but more importantly, they predict the patient’s clinical outcome and determine whether the patient can benefit from nutritional support, thereby, guiding their rational applicability in clinical settings (89). Understanding the patient’s nutritional status and timely implementation of nutritional therapy can improve the patient’s quality of life and reduce their risk of malnutrition (90). In recent years, imaging technology has rapidly advanced and also gained popularity in the field of nutritional assessment. CT scan can more directly and objectively assess the body’s skeletal muscle and fat content. In addition, it measures the CT value of muscles in an area that indirectly reflects the density of skeletal muscle. Therefore, the content and density of skeletal muscle as new indicators for evaluating nutritional status have attracted widespread scientific attention (91). For the treatment of ovarian cancer in, always we pay too much attention to quality assessment of the operation itself, but most are late ovarian cancer patients, patients constitution is poor, poor nutritional status, therefore, we should as soon as possible before surgery for patients with nutritional screening, such as we mentioned earlier NRS, PG-SGA score, etc., if the malnutrition, to correct as soon as possible, in addition. It is also intuitively important to assess the inflammatory status of muscle mass and function, as well as CRP and other inflammatory systems. Inflammatory responses can promote tumor progression through multiple pathways. Table 3 shows that the baseline status of city-wide inflammation can be used to predict disease-free survival and total mortality in ovarian cancer patients (Table 3). Of course, these studies also have limitations. There are many single-center retrospective studies, and there is a certain risk of bias. The critical values of each indicator of inflammation are not used, and the accuracy and sensitivity cannot meet the needs of clinical biomarkers. However, a “gold standard” is still lacking as the currently commonly used screening tools have their distinct characteristics. We believed that there was a very strong association within these indicators, both within nutritional status, inflammatory indicators, and between the two categories. Because inflammatory state induces catabolism and high protein consumption, with subsequent muscle loss (91). However, as we showed in Table 3, all these inflammatory indicators have some significance in the prognostic guidance of ovarian cancer (Table 3). However, their optimal cut-off values were different in the different cohorts. Thus, more forward-looking joint index screening approaches need to be developed in the future. The use of a variety of scores and a combination of the nutritional-related inflammation and muscle indicators are currently recommended to screen the nutritional status of patients more accurately with ovarian cancer. Future large-scale prospective studies, including ethnic, regional, and long-term follow-up, are needed to determine which markers are of greater prognostic value. This would further enable the formulation of a corresponding reasonable nutritional support regime. Finally, early detection and controlling of the progression of the disease are crucial to reducing its complications, improving the patients’ quality of life, and shortening their length of hospital stay.

**TABLE 3:** Relationship between inflammatory markers and prognosis.

	Author	*n*	Stage	Mean age	Index significance
**NLR**					
1	Medina Fernández et al. (55)	122	NA	55.8	NLR ≥ 8 from the beginning, and after having a clear fall in NLR, start exhibiting rising values should have a potential infective complication.
2	Zhou et al. (56)	370	III, *n* = 370	54.3	NLR > 3.08 had shorter PFS (16.9 vs. 19.5 months, HR = 1.3, 95% CI = 1.03–1.63, *P* = 0.022) and OS (33.5 vs. 46.8 months, HR 1.3, 95% CI = 1.01–1.66, *P* = 0.001).
3	Feng et al. (57)	875	(I, II), *n* = 75; (III, IV), *n* = 800	NA	A high NLR (≥ 3.24) was associated with reduced PFS (*P* < 0.001) and OS (*P* < 0.001).
4	Salman et al. (58)	111	IIIC, *n* = 75; IV, *n* = 801	63.3	NLR ≥ 6.0 was associated with significantly worse OS (*P* < 0.05).
5	Williams et al. (59)	519	NA	NA	Higher NLR was associated with significantly worse OS (*P* = 0.003).
6	Sanna et al. (60)	161	IIIC, *n* = 47; IVA, *n* = 76; IVB, *n* = 38	57	NLR > 1.58 had shorter PFS (10 vs. 24 months, HR = 9.3, 95% CI = 4.9–17.7, *P* < 0.0001).
7	Marchetti et al. (61)	397	(I, II), *n* = 136; (III, IV), *n* = 126	43.4	NLR < 4 had a significant 7-month increase in mPFS (26 vs. 19 months, *P* = 0.009).
8	Wu et al. (62)	262	(I, II), *n* = 136; (III, IV), *n* = 127	43.4	dNLR ≤ 2.11, distinguish ovarian cancer from benign ovarian disease (*P* < 0.001); dNLR ≤ 1.9, distinguish ovarian cancer from healthy controls
**PLR**					
1	Asher et al. (66)	235	I, *n* = 55; II, *n* = 28; III, *n* = 107; IV, *n* = 34; missing, *n* = 11	62	PLR < 300 had longer OS (37.4 vs. 14.5 months, *P* < 0.001)
2	Badora-Rybicka et al. (67)	315	I, *n* = 61; II, *n* = 30; III, *n* = 186; IV, *n* = 38	54	PLR < 62.31 had longer PFS (AUC: 0.665, 95% CI = 0.59–0.73, *P* < 0.0001); PLR < 129.78 had longer OS (AUC: 0.610, 95% CI = 0.55–0.67, *P* = 0.0008).
3	Raungkaewmanee et al. (68)	166	(I, II), *n* = 88; (III, IV), *n* = 78	53	PLR ≥ 200 had shorter PFS (*P* = 0.003) and OS (*P* = 0.002)
4	Zhang et al. (69)	190	I, *n* = 22; II, *n* = 31; III, *n* = 128; IV, *n* = 9	50.6	PLR > 203 had shorter PFS (11 vs. 24 months, *P* < 0.001) and OS (28 vs. 64 months, *P* < 0.001)
**LMR**					
1	Yang et al. (76)	364	(I, II), *n* = 52; (III, IV), *n* = 312	NA	LMR ≥ 3.84 had longer mPFS (88 vs. 56 months, *P* < 0.01) and mOS (100 vs. 69 months, *P* < 0.01)
2	Kwon et al. (77)	109	(I, II), *n* = 64; (III, IV), *n* = 45	50	LMR ≥ 4.2 had longer 5-year PFS (76.2 vs. 39.8%, *P* = 0.003) and OS rate (90.1 vs. 50.6%, *P* < 0.001)
3	Eo et al. (78)	234	(I, II), *n* = 97; (III, IV), *n* = 137	54	LMR > 2.07 had longer 5-year PFS (40.0 vs. 62.5%, *P* < 0.0001) and OS rate (42.2 vs. 67.2%, *P* < 0.0001)
**CAR**					
1	Komura et al. (84)	308	(I, II), *n* = 166; (III, IV), *n* = 144	NA	CRP/Alb > 0.048 had shorter OS (HR = 2.35; 95% CI, 1.30–4.48; *P* = 0.0044)
2	Liu et al. (85)	200	I, *n* = 25; II, *n* = 33; III, *n* = 107; IV, *n* = 35	53	Cut-off value = 0.68; CRP/Alb was associated with a more advanced tumor stage (*P* = 0.001), fewer patients with ideal cytoreductive surgery (*P* = 0.049), the presence of ascites (*P* = 0.009) and higher serum CA-125 level (*P* = 0.002).
**GPS**					
1	Sharma et al. (86)	154	III, *n* = 109; IV, *n* = 43	63.3	OS (months): GPS = 0, 40.9 (29.9–51.9); GPS = 1, 27.5 (23.3–31.8); GPS = 2, 22.4 (12.1–32.6); *P* = 0.02
2	Omichi et al. (87)	216	I, *n* = 87; II, *n* = 15; III, *n* = 88; IV, *n* = 26	61	The higher the GPS score, the shorter the OS and PFS (all *P* < 0.001)
3	Zhu et al. (88)	672	III, *n* = 564; IV, *n* = 108	55	The higher the GPS score, the shorter the OS and PFS (all *P* < 0.001)

*NA, not available.*

## Author Contributions

JM and YW: topic planning, manuscript development, and writing. CJ and LC: material collection and sorting. JC and DL: critical revision and supervision of the manuscript. All authors contributed to the article and approved the submitted version.

## Conflict of Interest

The authors declare that the research was conducted in the absence of any commercial or financial relationships that could be construed as a potential conflict of interest.

## Publisher’s Note

All claims expressed in this article are solely those of the authors and do not necessarily represent those of their affiliated organizations, or those of the publisher, the editors and the reviewers. Any product that may be evaluated in this article, or claim that may be made by its manufacturer, is not guaranteed or endorsed by the publisher.
